# Management of Laparoscopic Adjustable Gastric Band Erosion: A Case Report

**DOI:** 10.7759/cureus.47718

**Published:** 2023-10-26

**Authors:** Taylor F Faust, Emma Schnittka, Michael B Steadman, Garrett M Cail, Bradley S Rice

**Affiliations:** 1 Department of Research, Alabama College of Osteopathic Medicine, Dothan, USA; 2 Department of Gastroenterology, Crestwood Medical Center, Huntsville, USA

**Keywords:** erosion, abdominal, stomach, laparoscopic, gastric band, bariatric

## Abstract

Gastric banding was one of the first operations to gain popularity within the field of bariatric surgery. This case details one patient’s presentation and subsequent management of gastric band erosion with the hope of guiding other physicians and supporting the decreased use of gastric banding.

The patient, a 61-year-old Caucasian female, presented to the bariatric clinic complaining of a multiyear history of epigastric pain and acid reflux, which was refractory to treatment with proton pump inhibitors. She had a history of laparoscopic adjustable gastric band (LAGB) placement in 2007. She was initially successful in achieving weight loss and maintained regular band adjustments but was lost to follow-up and regained a body mass index (BMI) of 41.59 kg/m^2^. Evaluation with upper gastrointestinal (GI) endoscopy was recommended and performed. This revealed a LAGB in its entirety with tubing within the gastric fundus. Removal with dual endoscopy and abdominal laparoscopy was recommended and scheduled. During attempts to remove the band using an endoscopic snare, significant difficulty was encountered. Ultimately, an endoscopic rat-tooth grasper was used to lyse the band and tubing into four sections for complete removal. The subcutaneous port of the band was successfully removed laparoscopically, and the patient was discharged from the operating room. She reported limited pain in the postoperative suite but was lost to follow-up regarding long-term symptom relief.

This report describes the presentation and management of one patient’s experience with a known complication of LAGB-band erosion. This complication necessitated two additional procedures with anesthesia and placed the patient at increased risk for esophageal perforation, complications related to sedation, and the development of abdominal adhesions. Her case aims to support the decreasing prevalence of LAGBs within bariatric surgery and hopes to guide other physicians challenged with the management of similar cases.

## Introduction

Bariatric surgery encompasses some of the fastest-growing procedures worldwide. With nearly two million patients undergoing bariatric surgery between 1993 and 2016, this surgical field continues to grow and evolve [1]. Gastric banding was one of the first operations to gain popularity in this realm, in which the band is around the cardia to minimize stomach size and therefore limit oral intake [2]. Laparoscopic adjustable gastric bands (LAGBs) are bound to subcutaneous ports, which can be injected with saline to periodically alter the bands’ diameter and degree of restriction [3]. While LAGB placement comprised 35.4% of all bariatric procedures in 2011 in the United States, their use was diminished to 0.9% by 2019 in the same location [4]. Such decline is likely explained by LAGB’s limited postoperative weight loss and high complication rate [5,6]. This case report describes the presentation and management of one patient’s experience with a known complication of LAGB-band erosion and hopes to guide physicians in the management of similar case presentations.

## Case presentation

A 61-year-old Caucasian female presented to the bariatric clinic complaining of a multiyear history of epigastric pain and acid reflux. Her symptoms were intermittent but present most days. She noted that pain and reflux were most prominent after eating large meals. While symptoms had been present for the past two to three years, she noted an increase in severity over the past six months. Reflux had not responded to treatment with pantoprazole or omeprazole. She denied fatigue, rapid weight changes, nausea, vomiting, diarrhea, and abdominal pain.

The patient noted that she had a LAGB placed in 2007. She was initially successful in achieving weight loss and maintained regular band adjustments but was lost to follow-up after five years. She was uncertain which band was placed or the volume currently within the band. Medical records from her previous surgeon could not be obtained. Her BMI was 41.5 kg/m^2^ in the office. In addition to LAGB placement, her surgical history was significant for cardiac stent placement, carpal tunnel repair, cesarean section, and knee replacement. Her medical history included obesity, diabetes, hypertension, coronary artery disease, hyperlipidemia, and osteoarthritis. Her medication list included metformin, pantoprazole, losartan, atorvastatin, insulin, duloxetine, amitriptyline, and sulindac. She noted an allergy to sulfa drugs.

The physical exam showed a 61-year-old female in no acute distress with no evidence of trauma to the head, neck, trunk, or extremities. The patient was obese with significant gynoid fat distribution. The abdomen was benign with few well-healed incisions from a previous laparotomy. Further evaluation with an upper GI endoscopy was discussed, and the patient consented to this procedure.

**Figure 1:**
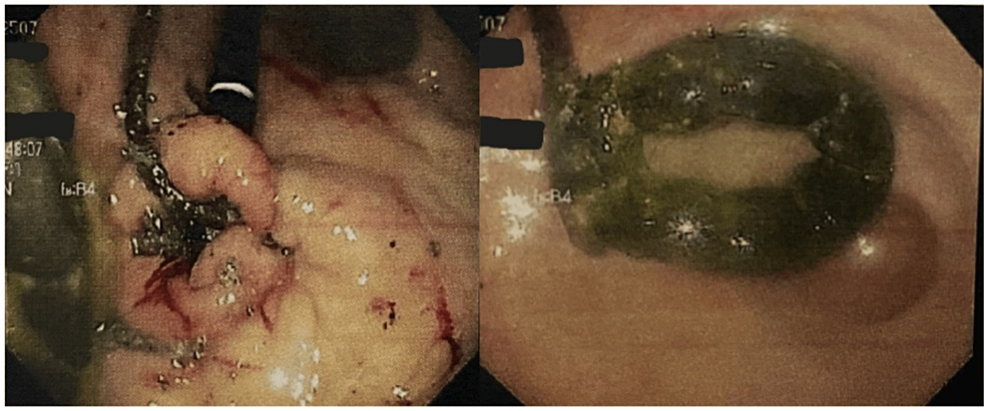
Endoscopic examination of the stomach with a benign tissue bridge (left) and an eroded gastric band (right)

Upper GI endoscopy revealed a LAGB with tubing within the gastric fundus (Figure 1). A 9-mm esophageal ulcer at the gastro-esophageal junction and an incidental bridge of tissue near the antrum were also noted (Figure 1). Biopsies of the gastric antrum were taken for *Helicobacter pylori* testing (negative). The results of the procedure were discussed with the patient. The patient chose to follow the surgeon's recommendation with the removal of the LAGB and port through a transabdominal incision with endoscopic scope.

**Figure 2:**
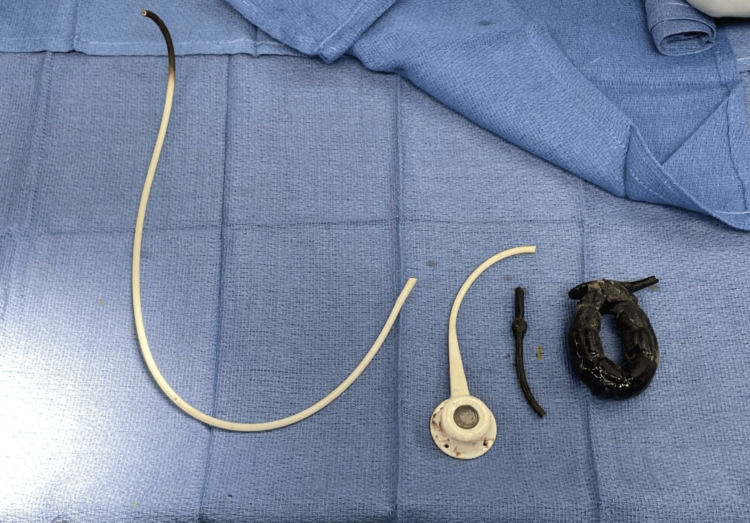
All removed components of the gastric band, tubing, and subcutaneous port as examined in the operating room.

Removal of the eroded LAGB began with a transverse incision overlying the adjustable port. After dissection through each layer of skin, the abdominal muscles, and surrounding fascia, the port was mobilized via lysis of surrounding sutures and the attached tubing and removed. The fascial and epidermal layers were then closed with a monocryl suture. An endoscope was then advanced orally, and the eroded LAGB and tubing were visualized. Initial attempts to encircle the band with a 27-mm endoscopic snare were unsuccessful. A rat-tooth grasper was then used to reposition the tubing. During the maneuver, a portion of the tubing was transected and was able to be extracted orally. Further repositioning with the rat-tooth grasper ligated another section of tubing, which was also removed. With only the LAGB remaining, a 33-mm snare was sufficient to surround the buckle of the specimen for extraction. All removed segments were examined for completeness (Figure 2). The stomach was inspected for any remaining fragments of band or injury before removal of the endoscope. The small gastrotomy remaining from port removal was expected to close naturally. The patient left the operating room in stable condition. She reported limited pain in the postoperative suite but was lost to follow-up regarding long-term relief.

## Discussion

In the United States alone, nearly two million patients underwent a bariatric procedure from 1993 to 2016 [1]. One of the first procedures within bariatric surgery was the LAGB, a circumferential device positioned around the cardia, which acts to decrease the amount of food that can be ingested by inducing early satiety thereby promoting weight loss. While LAGB placement comprised 35.4% of bariatric surgical procedures in 2011, their use was diminished to 0.9% by 2019 [2]. This decline is likely explained by the failure to achieve an adequate decrease in excess weight loss [5,6]. While the alternative bariatric procedures of laparoscopic sleeve gastrectomy (LSG) and Roux-en-Y gastric bypass (RYGB) show an average decrease of 43.5% and 50.7% in excess weight loss from baseline, respectively, LAGB patients averaged only 14.9% loss after 10 years [7]. As seen in this case, patients may initially lose weight only to regain it later [6,8].

The procedure’s complications, the need for continuous adjustments with close follow-up, and the necessity to remove the band in the long term may explain the reduction in LAGB placement. LAGBs have an estimated 5.8% complication rate [9,10]. Common complications include infection, abscess formation, perforation, peritonitis, and migration of the band to other parts of the gastrointestinal tract. While the prevalence of band erosion varies by source, one study found that of 865 patients undergoing LAGB placement, 18 were diagnosed with erosions postoperatively (1.96%). Of these, 10 occurred within the first year, with a median occurrence at seven months [11]. Other studies estimate an occurrence of erosion from 1.6% to 3% [12].

Band erosion can present with various symptoms, though the loss of appetite restriction and epigastric pain were two of the most common complaints reported in 42% and 29% of patients, respectively [13]. Other symptoms included recurrent port infections, regurgitation/reflux, fever, nausea/vomiting, anemia, back pain, and splenic abscess. However, 19% of patients with gastric band erosion were asymptomatic [10,13,14]. While symptoms of band erosion can be problematic, eroded bands can be safely removed endoscopically under circumstances where the buckle of the band can be visualized [13,15]. However, as seen in this case, some difficulty with removal can occur due to varying band sizes and degrees of erosion.

LAGB’s limited postoperative weight loss and high complication rate may raise the question of whether LAGBs should be utilized in modern bariatric surgery. However, it is essential to note the potential benefits of this procedure. Compared to LSG and RYGB, band placement is much less invasive, avoiding permanent removal of large portions of the stomach. Additionally, the mortality of this procedure is significantly lower than that of other bariatric surgeries (0.03% vs. 0.05% for LSG and 0.09% for RYGB) [16]. Also, failed LAGBs can usually be converted to other bariatric procedures, including RYGB and sleeve gastrectomy [17]. For these reasons, LAGBs maintain FDA approval and endorsement from the American Society of Metabolic and Bariatric Surgery (ASMBS), though they have lost favor as a first-line surgical intervention [4,5]. While not every case of gastric band erosion can be prevented, regular follow-up and monitoring of band function/adjustments can reduce this risk and promote bariatric surgery’s goal of weight loss and the reduction of complications secondary to metabolic disorders. Erosion-specific complications need to be further understood to better prevent, assess, and intervene when issues arise due to gastric bands.

This single case is an example of a less favorable outcome than what is currently featured in the literature. Presented by substantial erosion and less-than-optimal weight loss, this is an example of a larger issue that has contributed in part to the regression of this procedure in clinical practice. This necessitates an increased need for further research featuring complications such as this.

## Conclusions

Bariatric surgery is one of the fastest-growing surgical fields in the United States and worldwide. Gastric banding was one of the first procedures utilized within this realm, though its use has decreased over time, likely due to low efficacy and associated complications. This report describes the presentation and management of one patient’s experience with a known complication of LAGB-band erosion. This case aims to explain and support the decreasing prevalence of LAGBs within bariatric surgery and hopes to guide other physicians challenged with removing eroded gastric bands.
